# Dietary Copper Intake and Risk of Parkinson’s Disease: a Cross-sectional Study

**DOI:** 10.1007/s12011-023-03750-9

**Published:** 2023-07-18

**Authors:** Zhaohao Zeng, Yanmei Cen, Lijiao Xiong, Guo Hong, Yu Luo, Xiaoguang Luo

**Affiliations:** 1 Department of Neurology, Shenzhen People’s Hospital (The Second Clinical Medical College, Jinan University; The First Affiliated Hospital, Southern University of Science and Technology), Shenzhen, 518001 Guangdong China; 2 The First Clinical Medical College of Jinan University, Guangzhou, 510632 Guangdong China; 3 Guangdong Provincial Clinical Research Center for Geriatrics, Shenzhen Clinical Research Center for Geriatrics, Shenzhen People’s Hospital (The Second Clinical Medical College, Jinan University; The First Affiliated Hospital, Southern University of Science and Technology), Shenzhen, China; 4 Department of Geriatrics, Shenzhen People’s Hospital (The Second Clinical Medical College, Jinan University; The First Affiliated Hospital, Southern University of Science and Technology), Shenzhen, 518001 Guangdong China

**Keywords:** Parkinson’s disease (PD), Dietary copper intake, Propensity score matching (PSM), The National Health and Nutritional Examination Surveys (NHANES)

## Abstract

Copper is an essential trace element for the human body. The epidemiological evidence for the association of dietary intake of copper with the risk of Parkinson’s disease (PD) is limited. We conducted an evaluation of the cross-sectional data gathered from the National Health and Nutrition Examination Surveys spanning from 2007 to 2018, which comprised a total of 17,948 participants. To discern the distinct characteristics of the participants, we performed a univariate analysis and utilized a 1:2 ratio propensity score matching method to minimize the effects of selection bias. We employed weighted univariate as well as three multivariate logistic regression models both prior to and following matching, with the aim of examining the association between dietary copper intake and PD risk. Finally, we used the restricted cubic spline (RCS) methodology in order to investigate possible non-linear relationships. Furthermore, subgroup analysis was undertaken to elicit further understanding concerning the association between copper intake and PD. A negative correlation resulted between dietary copper intake and PD risk in both univariate and multivariate logistic regression models, prior to and following matching. Our findings demonstrate that there is a nonlinear, dose-dependent relationship between copper intake and PD, according to our RCS analysis. In subgroup analysis, copper intake was identified as an important protective factor for individuals who were non-Hispanic White, unmarried, and had completed higher education. Dietary copper intake was associated with the risk of PD. Supplementation of dietary copper may have potentially beneficial effects.

## Introduction

Parkinson’s disease (PD) is a progressive neurological disorder characterized by the degeneration of dopaminergic neurons [1]. At present, it is believed that the pathogenesis of PD is the result of the interaction between genetic factors and the environment [2]. Epidemiological studies have shown that there is a clear association between the occurrence of PD and exposure to some heavy metals such as lead, mercury, copper, and zinc [3]. After entering the body, these trace metals cross the blood-brain barrier and disrupt the redox equilibrium, causing free radical formation and reducing antioxidant enzyme levels [3, 4]. They may inhibit mitochondrial ATP synthesis and impair metabolic processes, which in turn destroy dopaminergic neurons [3]. However, they are equally indispensable trace elements involved in the proper functioning of many cellular enzymes and proteins [5–8]. In conclusion, the imbalance of heavy metal levels is closely related to the pathogenesis of PD.

A large number of enzymes rely on copper as a cofactor to function [9]. Copper is also essential for metabolism in the body [9]. Moreover, copper plays a key role in mitochondrial electron transport and is an essential cofactor for oxidoreductases [10, 11]. Copper, on the other hand, is a low-toxic metal, and excessive amounts will damage the nervous system [10]. It contributes to the pathological process of PD [10, 12, 13]. A debate exists regarding the correlation between copper concentration and PD pathogenesis. In some theories, excessive copper toxicity contributes to PD pathology [14], while some studies have found that copper deficiency contributes to PD pathology [15–17]. It has been demonstrated that PD patients have altered copper levels in their brains and serums [16, 18, 19]. A meta-analysis confirmed the presence of copper deficiency in the substantia nigra of patients with PD; however, no significant difference was observed in the levels of copper in their cerebrospinal fluid and serum [20]. Although a few epidemiological studies have suggested that there is no association between dietary copper intake and the risk of PD [21, 22], as mentioned above, there is a subtle association between dietary copper intake and PD, and the relationship between copper and PD risk is worthy of further investigation.

Given these facts, there is a requirement to clarify the associations between dietary copper intake and the risk of PD. Here, we conducted a cross-sectional study based on the National Health and Nutrition Examination Survey (NHANES) database aiming to evaluate dietary intake of copper and the risk of PD, which can be used as a reference for the prevention of PD.

## Methods

### Database and Survey Populations

**Fig. 1:**
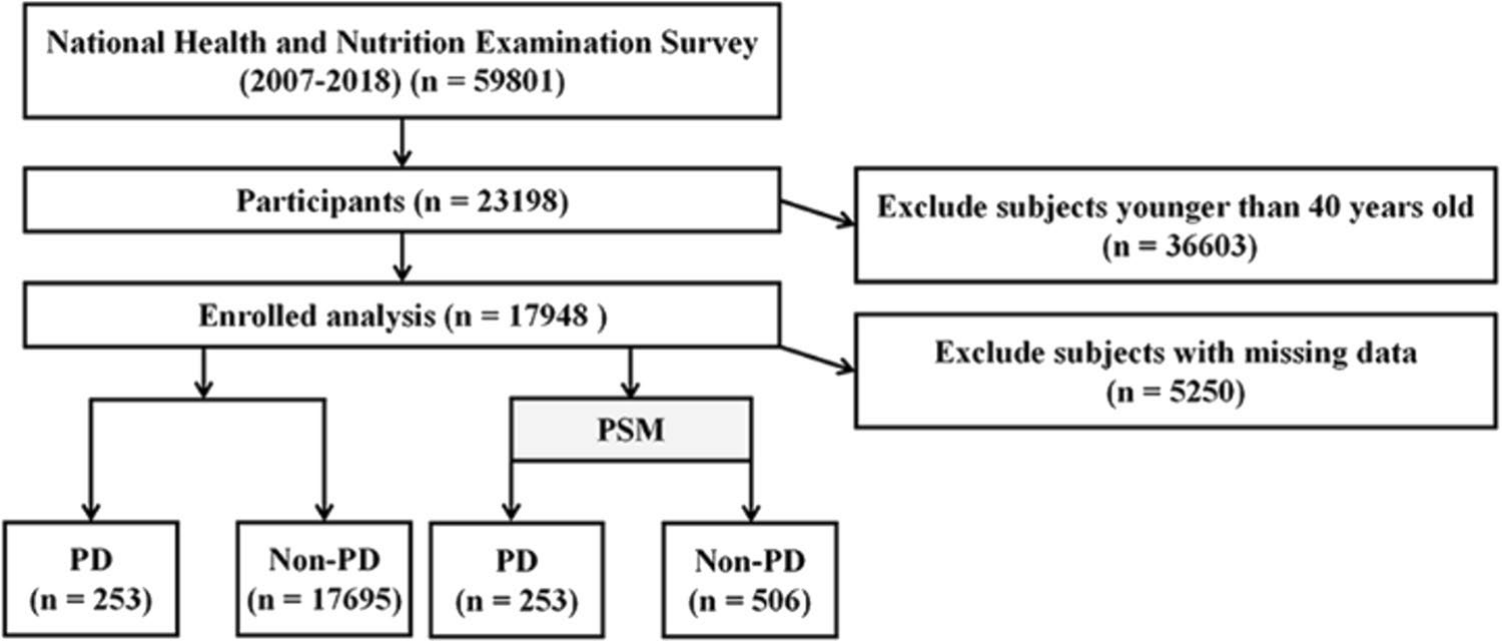
Flow chart of study inclusion. Flowchart of the participants’ selection from NHANES 2007–2018

Data for this study were obtained from the National Health and Nutrition Examination Survey website (https://www.cdc.gov/nchs/nhanes/index.htm). We constructed a dataset for this study by using publicly available NHANES responses from 2007 to 2018. A total of 17,948 NHANES participants over 40 years old were included in this study. The entire data integration process is shown in Fig. 1.

### Assessment of Parkinson’s Disease

In the NHANES database, combined with previous literature [23–25], participants with Parkinson’s disease were identified by using “ANTIPAKINSON AGENTS”; this determination was made based on the responses to prescription medication questions. Due to the limitations of medications and codes included in the NHANES, an individual had to be receiving treatment for PD to be classified as having it, and others were identified with non-Parkinson’s disease.

### Dietary Copper Intake

Copper intake data was obtained from the NHANES database, and participants were asked to recall their copper intake twice in 24 h. During the first recall interview, the respondent completed a test at the NHANES Mobile Testing Center, while respondents were asked to complete a telephone interview 3–10 days in the second recall interview. In our study, to calculate dietary copper intake, we averaged data from two dietary recalls; otherwise, the single diet data will be deleted.

### Covariates Assessment

The main covariates were demographic characteristics and chronic comorbidities. Demographic characteristics including age, gender, race, education level, and marital status were reported by interviewees. Chronic comorbidities included diabetes, coronary heart disease, hypertension, stroke, and viral hepatitis, which were diagnosed based either by the doctor or by a self-report questionnaire. Weight and height were measured by well-trained health technologists, and BMI was calculated as weight (kg) divided by height squared (m^2^).

### Statistical Analysis

**Fig. 2:**
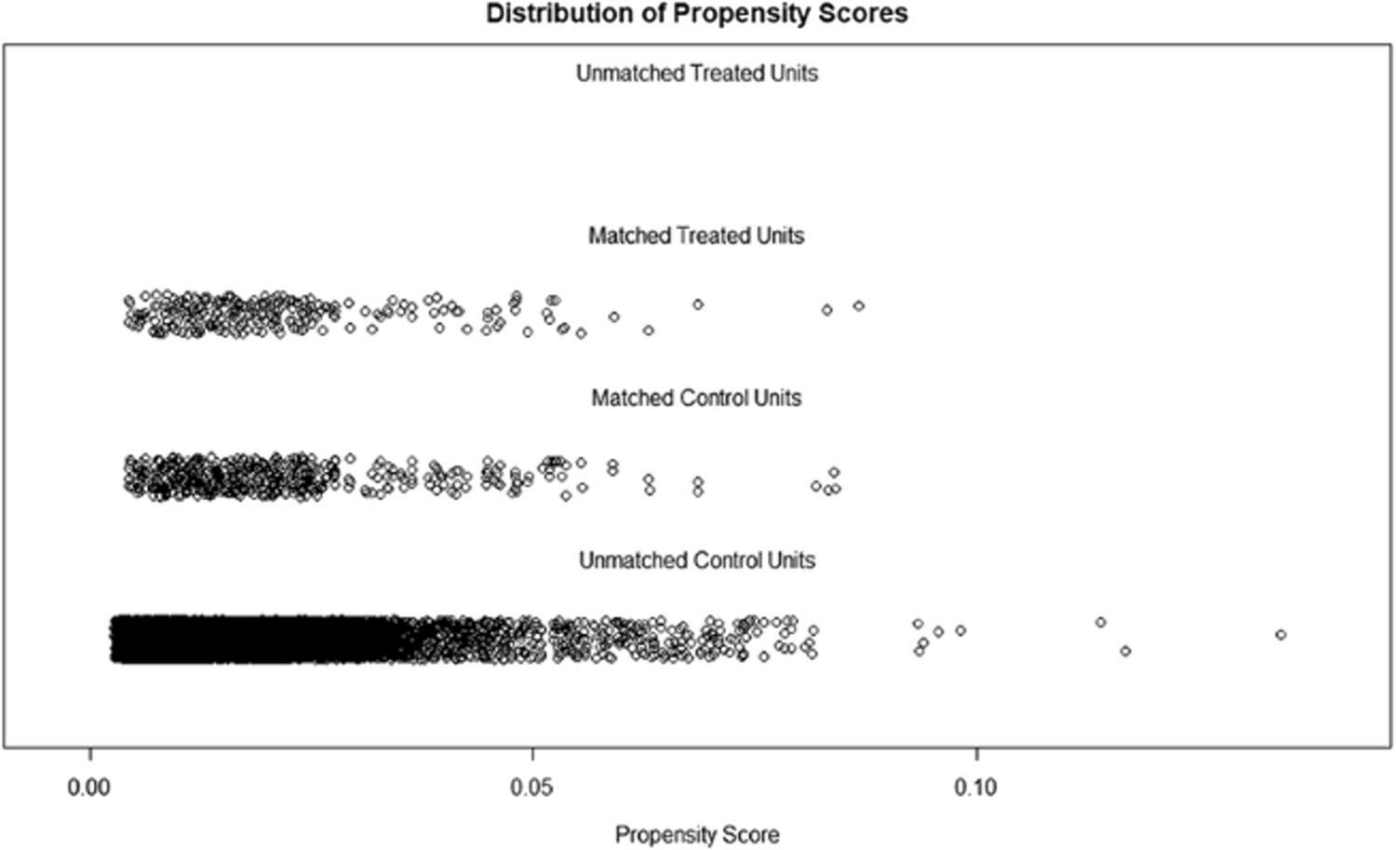
“Jitter” diagram of PSM. Before undergoing propensity score matching (PSM) at a 1:2 ratio, unmatched treated units, and unmatched non-treated units represented the distribution of individuals in the PD and non-PD groups, respectively. After matching, the two groups were represented by matched treated units and matched non-treated units, respectively. The figure illustrates the balanced distribution of individuals in the matched groups following PSM

Data extraction and analyses were performed with the use of the “nhanesR” package of R software (4.2.1). Categorical variables are expressed as frequency and percentages, while continuous variables are expressed as means and standard deviations (SD). The analysis was based on the non-parametric Mann-Whitney *U* test or the *t*-test for continuous variables. During the comparisons of categorical variables, chi-square tests and Fisher’s exact tests were used. Propensity score matching (PSM) is a method that can reduce selection bias to strengthen causal arguments in observational studies (as shown in Fig. 2). A 1:2 rule is used in PSM to perform nearest-neighbor matching. An analysis of the relationship between dietary copper intake and PD risk was conducted using weighted univariate and three multivariate logistic regression models and possible non-linear relationships are determined by restricted cubic splines (RCS). Finally, we conducted subgroup analyses according to age, gender, race, marital status, and education level.

## Results

### Characteristics of Included Participants Before Matching by PD

As shown in Table 1, there were significant differences in gender (*p* = 0.027), age (*p* = 0.003), race (*p* = 0.017), coronary heart disease (*p* = 0.038), and stroke (*p* < 0.0001) between the PD and non-PD groups. It seems that the participants were older than those of the non-PD group (61.262±1.103) vs. 57.938(±0.174) years). Most importantly, there was a significant difference in dietary copper intake between PD and non-PD with 1.069 (±0.047) and 1.276 (±0.014) (*p*<0.0001).

**Table 1:** Baseline characteristics of participants with or without PD

Characters	Total	Non-Parkinson	Parkinson	*p*-value
Gender				**0.027**
Male	8571 (47.757)	8458 (46.819)	113 (35.969)	
Female	9376 (52.243)	9236 (53.181)	140 (64.031)	
Age	57.983 (0.175)	57.938 (0.174)	61.262 (1.103)	**0.003**
Race				**0.017**
Non-Hispanic White	8103 (45.15)	7937 (71.541)	166 (81.310)	
Non-Hispanic Black	3956 (22.043)	3918 (10.478)	38 (8.874)	
Mexican American	2364 (13.172)	2342 (6.333)	22 (3.382)	
Other race	3524 (19.636)	3497 (11.649)	27 (6.434)	
Marital				0.060
Married	13,062 (72.781)	12,880 (74.529)	182 (64.728)	
Unmarried	4885 (27.219)	4814 (25.471)	71 (35.272)	
Education				0.089
Lower high school	4464 (24.873)	4390 (15.212)	74 (22.804)	
High school	4158 (23.168)	4100 (23.535)	58 (25.054)	
Over high school	9325 (51.959)	9204 (61.253)	121 (52.142)	
BMI	29.532 (0.097)	29.518 (0.097)	30.564 (0.557)	0.062
Copper	1.273 (0.014)	1.276 (0.014)	1.069 (0.047)	**< 0.0001**
Coronary heart disease				**0.038**
No	16,846 (93.865)	16,617 (94.624)	229 (89.155)	
Yes	1101 (6.135)	1077 (5.376)	24 (10.845)	
Diabetes				0.215
No	12,306 (68.569)	12,154 (75.277)	152 (69.177)	
Pre-diabetes	1133 (6.313)	1116 (5.635)	17 (6.775)	
Diabetes	4508 (25.118)	4424 (19.088)	84 (24.048)	
Hypertension				**0.004**
No	7769 (43.289)	7692 (49.248)	77 (34.917)	
Yes	10,178 (56.711)	10,002 (50.752)	176 (65.083)	
Stroke				**< 0.0001**
No	16,926 (94.311)	16,710 (95.664)	216 (84.270)	
Yes	1021 (5.689)	984 (4.336)	37 (15.730)	
Viral hepatitis				0.475
No	17,449 (97.225)	17,206 (97.571)	243 (96.759)	
Yes	498 (2.775)	488 (2.429)	10 (3.241)	

Data are presented as *N*% (*χ*^2^ test) or mean (SD) (independent *t-*test). *p < 0.05; **p < 0.01; ***p < 0.001

The use of bold font indicates that the corresponding *p*-values are less than 0.05, signifying statistical significance

### Characteristics of Participants After Matching by PD

Our study established a comparable control group using closest-neighbor PSM (1:2) to help confirm the association between dietary copper intake and the risk of Parkinson’s disease. After PSM, data sets with 253 participants in the PD group and 506 participants in the non-PD group were constructed. Table 2 shows that dietary copper intake (*p* = 0.001) was also significantly different between the PD group and the non-PD group after matching, and other covariates were not statistically different (Table 2).

**Table 2:** Characteristics of participants after matching by PD

Characters	Total	Non-Parkinson	Parkinson	*p*-value
Gender				0.689
Male	328 (43.215)	215 (43.192)	113 (35.969)	
Female	431 (56.785)	291 (56.808)	140 (64.031)	
Age	62.052 (0.594)	62.383 (0.641)	61.262 (1.103)	0.35
Race				0.984
Non-Hispanic White	500 (65.876)	334 (86.016)	166 (81.310)	
Non-Hispanic Black	113 (14.888)	75 (5.611)	38 (8.874)	
Mexican American	68 (8.959)	46 (2.791)	22 (3.382)	
Other race	78 (10.277)	51 (5.581)	27 (6.434)	
Marital				0.55
Married	563 (74.177)	381 (74.463)	182 (64.728)	
Unmarried	196 (25.823)	125 (25.537)	71 (35.272)	
Education				0.986
Lower high school	226 (29.776)	152 (19.764)	74 (22.804)	
High school	173 (22.793)	115 (20.500)	58 (25.054)	
Over high school	360 (47.431)	239 (59.736)	121 (52.142)	
BMI	30.409 (0.392)	30.344 (0.488)	30.564 (0.557)	0.758
Copper	1.235 (0.037)	1.293 (0.048)	1.096 (0.036)	**0.001**
Coronary heart disease				0.705
No	680 (89.592)	451 (88.165)	229 (89.155)	
Yes	79 (10.408)	55 (11.835)	24 (10.845)	
Diabetes				0.957
No	455 (59.947)	303 (70.250)	152 (69.177)	
Pre-diabetes	48 (6.324)	31 (4.944)	17 (6.775)	
Diabetes	256 (33.729)	172 (24.806)	84 (24.048)	
Hypertension				0.878
No	227 (29.908)	150 (36.458)	77 (34.917)	
Yes	532 (70.092)	356 (63.542)	176 (65.083)	
Stroke				0.836
No	652 (85.903)	436 (89.776)	216 (84.270)	
Yes	107 (14.097)	70 (10.224)	37 (15.730)	
Viral hepatitis				0.759
No	732 (96.443)	489 (97.087)	243 (96.759)	
Yes	27 (3.557)	17 (2.913)	10 (3.241)	

Data are presented as *N*% (*χ*^2^ test) or mean (SD) (independent *t*-test). *p < 0.05; **p < 0.01; ***p < 0.001

The use of bold font indicates that the corresponding *p*-values are less than 0.05, signifying statistical significance

### Correlation Analysis of Copper Intake and PD Before and After Matching

#### Analysis of Univariate Logistics Regressions of Copper Intake and Physical Activity Before and After Matching

Table 3 shows that age and BMI were positively correlated with PD occurrence, and the effect value OR and 95% confidence interval were 1.024 (1.009, 1.040) (*p*=0.003) and 1.022 (1.001, 1.044) (*p*=0.043). Females were more likely to develop PD than males, with an OR of 1.567 (1.049, 2.342) (*p* = 0.029). Coronary heart disease, hypertension, and strokes were associated with a higher risk of PD than those without coronary heart disease, hypertension, and strokes, with odds ratios and 95% confidence intervals of 2.141 (1.027, 4.464) (*p*=0.042), 1.809 (1.202, 2.722) (*p*=0.005), and 4.119 (2.351, 7.214) (*p*<0.0001), respectively. Most importantly, we found that copper intake was negatively associate with the occurrence of PD (OR: 0.574 (0.404, 0.816) (*p*=0.002) and 0.498 (0.337, 0.735) (*p*<0.001)) before and after matching, respectively (Table 3).

**Table 3:** Univariate logistics regression analysis of the association between copper intake and PD before and after matching

Characters	Unmatching		Matching	
	OR (95% CI)	*p*-value	OR (95% CI)	*p*-value
Gender				
Male	Ref	Ref	Ref	Ref
Female	1.567 (1.049, 2.342)	**0.029**	1.353 (0.852, 2.150)	0.197
Age	1.024 (1.009, 1.040)	**0.003**	0.992 (0.976, 1.009)	0.350
Race				
Non-Hispanic White	Ref	Ref	Ref	Ref
Non-Hispanic Black	0.745 (0.420, 1.323)	0.312	1.673 (0.934, 2.997)	0.083
Mexican American	0.470 (0.270, 0.817)	**0.008**	1.282 (0.651, 2.525)	0.468
Other race	0.486 (0.271, 0.870)	**0.016**	1.219 (0.555, 2.678)	0.617
Marital				
Married	Ref	Ref	Ref	Ref
Unmarried	1.594 (0.977, 2.603)	0.062	1.589 (0.903, 2.797)	0.107
Education				
Lower high school	Ref	Ref	Ref	Ref
High school	0.710 (0.381, 1.322)	0.277	1.059 (0.511, 2.193)	0.876
Over high school	0.568 (0.321, 1.004)	0.052	0.757 (0.397, 1.441)	0.392
BMI	1.022 (1.001, 1.044)	**0.043**	1.004 (0.979, 1.030)	0.760
Copper	0.574 (0.404, 0.816)	**0.002**	0.498 (0.337, 0.735)	**<0.001**
Coronary heart disease				
No	Ref	Ref	Ref	Ref
Yes	2.141 (1.027, 4.464)	**0.042**	0.906 (0.416, 1.976)	0.802
DM				
No	Ref	Ref	Ref	Ref
Pre-diabetes	1.308 (0.675, 2.536)	0.422	1.392 (0.566, 3.424)	0.468
Diabetes	1.371 (0.958, 1.961)	0.084	0.984 (0.643, 1.507)	0.942
Hypertension				
No	Ref	Ref	Ref	Ref
Yes	1.809 (1.202, 2.722)	**0.005**	1.069 (0.660, 1.732)	0.783
Stroke				
No	Ref	Ref	Ref	Ref
Yes	4.119 (2.351, 7.214)	**<0.0001**	1.639 (0.849, 3.163)	0.139
Viral hepatitis				
No	Ref	Ref	Ref	Ref
Yes	1.346 (0.590, 3.068)	0.476	1.117 (0.328, 3.805)	0.858

*p < 0.05; ** *p* < 0.01; *** *p* < 0.001

The use of bold font indicates that the corresponding* p*-values are less than 0.05, signifying statistical significance

#### Multivariable Logistics Regression Analysis of the Relationship Between Copper Intake and PD Before and After Matching

We utilized three logistic regression models (Table 4) to investigate the connection between copper intake and PD. Model 1 was a crude model that did not adjust for any covariates, while model 2 adjusted for age, gender, race, and BMI. Model 3 incorporated adjustments for age, gender, race, education level, BMI, marital status, coronary heart disease, diabetes, hypertension, stroke, and viral hepatitis. In both pre- and post-matching scenarios, our findings indicate that all models displayed significant disparities. Our results suggest that daily copper intake is closely linked to PD, with OR values and 95% confidence intervals of 0.574 (0.404, 0.816), 0.627 (0.437, 0.900), and 0.696 (0.498, 0.973) (*p*<0.05) before matching, respectively. This implies that an increase in dietary copper intake acts as a protective factor against PD and is negatively associated with the risk of PD. Moreover, a significant correlation between copper intake and PD persisted even after matching was conducted.

**Table 4:** Multivariable logistics regression analysis of the association between copper intake and PD before and after matching

Model	Unmatching			Matching		
	OR (95% CI)	*p*-value	*p* for trend	OR (95% CI)	*p*-value	*p* for trend
Model 1	0.574 (0.404, 0.816)	0.002	0.002	0.498 (0.337, 0.735)	<0.001	<0.001
Model 2	0.627 (0.437, 0.900)	0.012	0.012	0.490 (0.322, 0.745)	0.001	0.001
Model 3	0.696 (0.498, 0.973)	0.034	0.034	0.498 (0.327, 0.759)	0.002	0.002

Model 1: no adjustments made for confounding factors. Model 2: adjustments made for age, gender, race, and BMI. Model 3: adjustments made for age, gender, race, education level, BMI, marital status, coronary heart disease, diabetes, hypertension, stroke, and viral hepatitis. *p < 0.05; **p < 0.01; ***p < 0.001

The use of bold font indicates that the corresponding* p*-values are less than 0.05, signifying statistical significance

#### Nonlinear Relationship Between Dietary Copper Intake and the Risk of PD

**Fig. 3:**
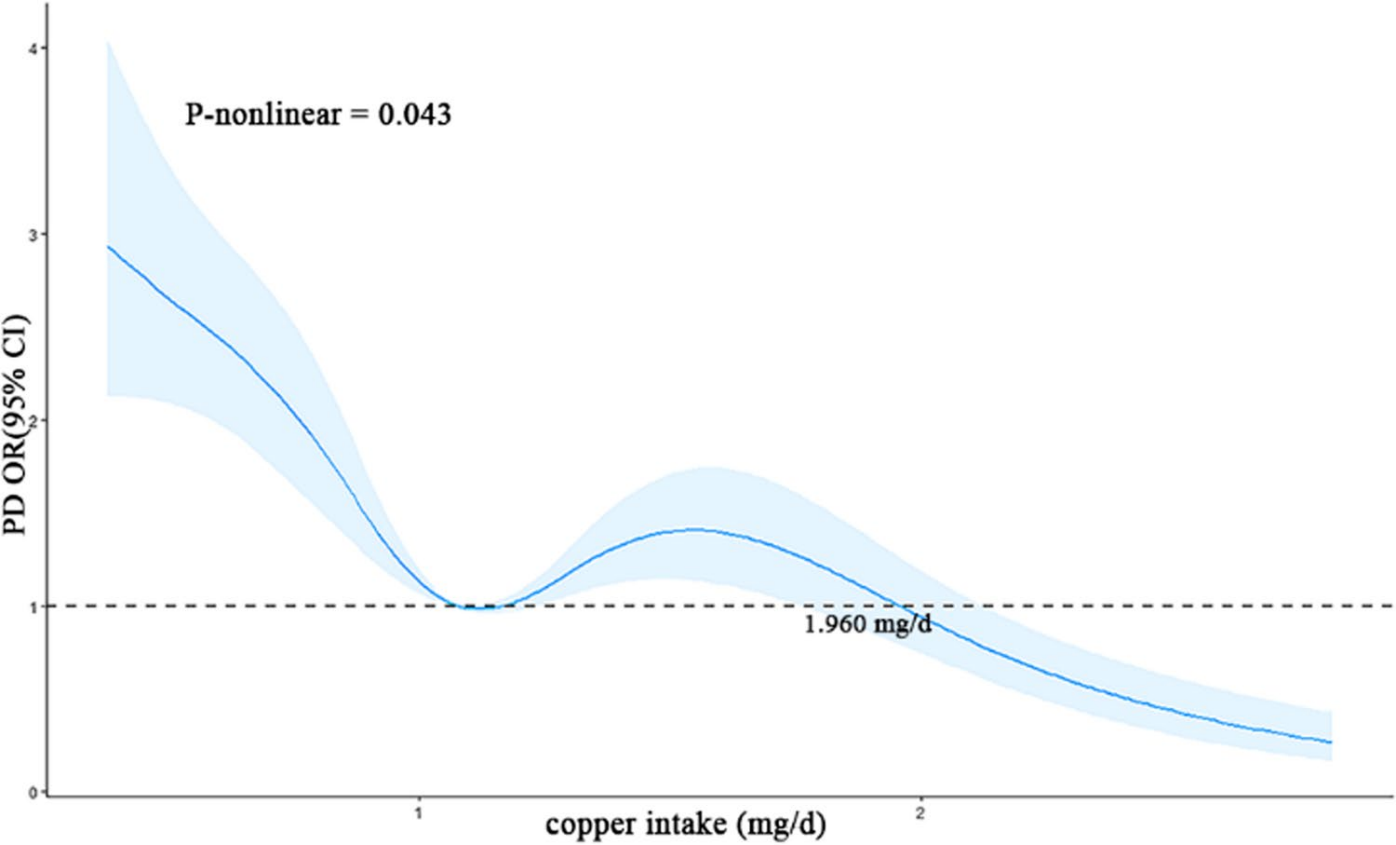
The RCS plot between copper intake and PD. RCS, restricted cubic spline. RCS plot between copper intake and PD after matching. The *x*-axis represents copper intake (mg/day), while the *y*-axis represents the odds ratio (OR) and the 95% confidence interval (CI) for PD (blue area). The dashed line indicates an OR of 1, which represents no association between copper intake and PD risk. The model adjusted by BMI, age, gender, race, marital status, education level, coronary heart disease, diabetes, hypertension, stroke, and viral hepatitis

Utilizing RCS, we generated a graphical depiction (Fig. 3) of the connection between dietary copper intake and PD. Our results indicate a non-linear relationship between dietary copper intake and PD risk (*p*=0.04) after matching had been performed. Specifically, our findings suggest that copper intake is a risk factor for PD when intake levels remain below 1.960 mg/day. However, the risk of developing PD shows a gradual decrease as copper intake surpasses 1.960 mg/day.

#### Subgroup Analysis Before and After Matching

To determine whether the relationship between copper intake and PD varied by age, gender, race, marital, and education, stratified analyses were employed. Based on subgroup analysis, copper intake was correlated with PD more strongly in 50–60-year-olds after matching, with odds ratios of 0.293 (0.133, 0.647), while were no significant differences before matching. Likewise, male, non-Hispanic White, unmarried, and over high school had a stronger correlation between copper intake and PD before matching with odds ratios of 0.548 (0.356, 0.844), 0.539 (0.349, 0.832), 0.390 (0.212, 0.715), and 0.529 (0.331, 0.846), while female, non-Hispanic White, unmarried, and over high school had a stronger correlation between copper intake and PD after matching with odds ratios of 0.443 (0.225, 0.874), 0.442 (0.267, 0.733), 0.277 (0.115, 0.667), and 0.522 (0.283, 0.964), respectively. There is an interaction between copper intake and marital status (*p* = 0.013) before PSM. In a general way, the consistent results before and after PSM matching are considered to be reliable. Therefore, copper intake was found to be a significant protective factor in people who were non-Hispanic White, unmarried, and over high school (Table 5).

**Table 5:** Subgroup analysis before and after matching

Characters	Unmatching			Matching		
	OR (95% CI)	*p*-value	*p* for interaction	OR (95% CI)	*p*-value	*p* for interaction
Age			0.683			0.118
40-50 year old	0.542 (0.284, 1.032)	0.062		0.243 (0.050, 1.166)	0.069	
50-60 year old	0.576 (0.295, 1.127)	0.106		0.293 (0.133, 0.647)	**0.004**	
60-70 year old	0.604 (0.319, 1.143)	0.119		0.404 (0.144, 1.134)	0.082	
>70 year old	0.772 (0.505, 1.178)	0.227		1.046 (0.519, 2.107)	0.898	
Gender			0.268			0.584
Male	0.548 (0.356, 0.844)	**0.007**		0.635 (0.363, 1.111)	0.109	
Female	0.771 (0.497, 1.196)	0.242		0.443 (0.225, 0.874)	**0.020**	
Race			0.164			0.252
Non-Hispanic White	0.539 (0.349, 0.832)	**0.006**		0.442 (0.267, 0.733)	**0.002**	
Non-Hispanic Black	0.788 (0.455, 1.364)	0.390		0.907 (0.331, 2.488)	0.802	
Mexican American	1.064 (0.944, 1.200)	0.305		0.747 (0.247, 2.263000e+00)	0.658	
Other race	0.910 (0.454, 1.825)	0.788		0.848 (0.335, 2.148)	0.757	
Marital			**0.013**			0.118
Married	0.784 (0.580, 1.059)	0.111		0.646 (0.390, 1.071)	0.089	
Unmarried	0.390 (0.212, 0.715)	**0.003**		0.277 (0.115, 0.667)	**0.006**	
Education			0.546			0.641
Lower high school	0.870 (0.619, 1.222)	0.416		0.709 (0.340, 1.478)	0.348	
High school	0.703 (0.223, 2.214)	0.543		0.465 (0.143, 1.510)	0.190	
Over high school	0.529 (0.331, 0.846)	**0.008**		0.522 (0.283, 0.964)	**0.038**	

Subgroup analysis for the association between copper intake and PD. Weighted univariate logistic regression was used for subgroup analysis. Adjustments were made for BMI, coronary heart disease, diabetes, hypertension, stroke, and viral hepatitis. *p < 0.05; **p < 0.01; ***p < 0.001

The use of bold font indicates that the corresponding *p*-values are less than 0.05, signifying statistical significance

## Discussion

Copper is an essential metal element for proper nervous system function. However, the exact role of metallic copper in PD remains unclear. Therefore, it is crucial from both clinical and societal perspectives to investigate the relationship between dietary copper and PD. In our study, we examined the correlation between dietary copper intake and PD, with multivariate logistic regression revealing a negative association between increased copper intake and PD risk. To minimize confounding variables’ influence, we utilized PSM analysis to account for differences between the PD and non-PD groups. After matching, RCS analysis demonstrated that the relationship between dietary copper intake and PD risk was non-linear.

Copper is an essential metal element for human bodily function, primarily obtained and supplemented through daily dietary intake via the small intestine [26]. Within the body, copper plays a pivotal role in activating various enzyme activities, cell physiological metabolism, oxidative phosphorylation, and electron transport [3, 5–7, 10]. Disruptions to copper homeostasis can lead to nervous system diseases [6, 8, 10], though its association with PD remains controversial. Some studies have suggested that copper concentrations in PD blood are reduced [15, 16, 18], while others have reported increased copper concentration in PD blood [27]. Additionally, meta-analyses have failed to identify significant differences in serum copper concentration between individuals with PD and those without [20].

The results of copper concentration in cerebrospinal fluid of PD are used to be controversial. An earlier study involving 24 PD patients and 34 controls found that PD patients had higher copper concentrations in their CSF [28]. Furthermore, by comparing 215 PD patients with 119 controls, no difference was found in CSF copper levels in PD patients [29]. The findings of a 2019 meta-analysis indicated that there was no significant difference in the levels of copper observed in the cerebrospinal fluid of patients with PD and those without the condition [20]. Interestingly, analysis of copper content in frozen sections of brain parenchyma revealed a significant reduction in copper content, ranging from 45 to 65%, in substantia nigra with severe PD lesions. This finding was further confirmed by electron microscopy imaging, which showed a significant reduction in copper content by 55% in neurons in locus coeruleus [19]. The meta-analysis also confirmed a significant reduction of copper levels in the substantia nigra of patients with PD [20]. The above evidence supports that the imbalance of copper homeostasis is closely related to PD. In fact, copper is involved in the neuropathological changes of PD. In vitro experiments showed that copper could directly bind to α-synuclein and induce its abnormal aggregation [12, 30]. In addition, it can directly participate in the oxidation of dopamine, resulting in oxidative damage to dopamine nerves and aggravating the pathological changes of PD [14, 31]. By increasing the expression of heat shock protein Hsp27 to bind the excess free copper ions, the neurotoxicity caused by copper imbalance can be alleviated to a certain extent. The interaction of α-synuclein with copper reduces the electrostatic repulsion between the negative charges in the polypeptide chain, the partial misfolded conformation of the peptide chain is more stable, and the tendency of α-synuclein to aggregate is increased [17]. Given the observed decrease of copper in the substantia nigra of individuals with PD, it appears that the mechanism of alpha-synuclein aggregation caused by an increase in copper is unlikely to occur in the PD brain. And some studies have shown the opposite. Copper also exerts an indirect regulatory effect on α-synuclein through other copper affinities proteins such as ATOX1 and Ctr1. In vitro experiments confirmed that these copper-avidity proteins could inhibit the folding and aggregation of α-synuclein and alleviate the pathological changes of PD [32, 33]. Besides, the reduced binding of the antioxidant enzyme SOD1 to copper can alter protein conformation, leading to a reduction in antioxidant activity [34]. Copper deficiency-induced abnormal folding of SOD1 is a potential mechanism that may contribute to the development of PD. Evidence of abnormal SOD1 folding and metal deficiency has been demonstrated in the brains of individuals with PD [35, 36]. As copper is almost supplemented by dietary intake, previous studies have also investigated the relationship between dietary copper intake and PD; however, the results showed no significant difference in dietary copper intake between PD and control populations [21, 37].

An analysis was conducted to determine the nonlinear relationship between variables and outcomes. According to RCS analysis, there appears to be a connection between dietary copper intake and PD. Specifically, when it comes to copper intake levels below or above 1.960mg/day, the effect of copper seems to be reversed. Within a certain range of change, dietary copper intake may be a risk factor for PD, but as intake levels increase beyond 1.960 mg/day, the risk of PD decreases. This complex and controversial relationship between copper and PD from dietary intake levels is further explained. To maintain healthy copper intake, it is crucial to control intake within a certain range. Our findings may provide new perspectives for health policymakers in preventing PD, suggesting that people consume more than 1.960 mg/day rather than less than 1.960 mg/day. Furthermore, subgroup analysis revealed that dietary copper intake served as a protective characteristic for individuals non-Hispanic White, unmarried, and with a high school education level. It is worth noting that excessive copper intake can lead to adverse effects such as liver failure. To ensure safety, the National Academy of Sciences recommends that the daily intake of copper should not exceed 10mg [38].

There are several advantages and implications to our study. First, this study is based on the sample survey of the American population, which has the characteristics of high credibility and strong representativeness. Second, by reducing selection bias, PSM strengthens the causal arguments in observational studies. Thirdly, through the use of RCS analysis, we were able to demonstrate the nonlinear associations between dietary copper intake and Parkinson’s disease, illuminating new insights that may be useful for health policymakers through the result of RCS curves and cutoff values. However, this study also has some limitations. First, NHANES relies on the use of anti-Parkinson’s drugs to define Parkinson’s disease patients, which may not accurately diagnose the condition. Additionally, other patient populations, such as those with a history of stroke or psychiatric illness, may also take anti-Parkinson’s drugs, potentially confounding the results in case studies. Second, due to the cross-sectional nature of the study, no causal inference can be made. The confounding factors selected for this study have limitations, and the influence of many unidentified confounders could not be completely excluded from this study. It is necessary to conduct more prospective clinical studies to clarify the relationship between copper intake and PD. In addition, the complex relationship between copper and PD also needs more basic research to clarify.

## Conclusions

In conclusion, this study suggests that a higher intake of dietary copper is associated with a lower risk of PD, and that the relationship between copper intake and PD risk is nonlinear. Given the increasing prevalence of PD and the potential neuroprotective effects of copper, this finding has implications for public health and clinical practice, as it suggests that maintaining adequate levels of dietary copper may be important for reducing the risk of PD. However, further research is needed to understand the underlying mechanisms and explore the potential benefits and risks of copper supplementation.

## Data Availability

Publicly available datasets were analyzed in this study. This data can be found here: https://www.cdc.gov/nchs/nhanes/index.htm.
